# Vector borne pathogens and host complement system: a tangled tale

**DOI:** 10.1186/1756-3305-7-S1-O9

**Published:** 2014-04-01

**Authors:** M Bhide, S Dolinska, E Bencurova

**Affiliations:** 1 Laboratory of biomedical microbiology and immunology, University of veterinary medicine and pharmacy, Komenskeho -73, Kosice, Slovakia

## 

Complement-mediated lysis of pathogens is the effective immune mechanism developed in the mammalian hosts, nevertheless, pathogens have also evolved complement evasion strategies to infect the hosts. Study of complement evasion mechanisms in various host help to understand many aspects like, pathogenesis, reservoir competence, association and host selectivity of vector-borne pathogens etc., and thus forms unique benchmarking to prevent the infection within the 'One Health' concept. The most effective complement evasion mechanisms employed by pathogens are: 1. binding of the host complement-regulatory/inhibitory proteins (CRPs) on their surface and 2. expression of CRP mimicking molecules. A comparative study of complement-pathogen interaction was performed, that included three different hosts (human as susceptible, cattle as resistant and sheep as intermediate susceptible host) and a repertoire of *Borrelia *(twelve species) and *Francisella *strains (three subspecies). Series of experiments like affinity ligand binding experiments, pull-down assays and mass spectrometry based protein identification revealed an array of surface proteins of *Borrelia *(37 proteins) and *Francisella *(6 proteins) that bind human factor H or C4BP or vitronectin to block complement activation. We also found that some stains of *Borrelia *possess human CD46 mimicking molecule, while some *Borrelia *and *Francisella *may express human CD59-homologous protein. Results shows that these vector-borne pathogens possess multiple proteins that can bind or mimic various CRPs and block human complement. It is noteworthy that, two proteins of *Francisella *(LVS, Tul4 strains) bound vitronectin only, whereas, only two proteins of *Borrelia *(of strains SKT-2 and DN127) bound ovine C4BP or factor H. It shows that both pathogens possess very few proteins that can bind ovine CRPs and insufficiently protect bacteria against ovine complement attack, which correlates with intermediate competence nature of sheep for both pathogens. None of the *Borrelia *and *Francisella *strains, except *B. coriaceae*, possessed factor H binding protein. This also correlates with the fact that only *B. coriaceae *effectively evade bovine complement system and infect cattle. This corresponds with non-competence nature of cattle for both pathogens. In summary, study of tangled association between surface proteins of pathogens, CRPs, complement evasion in various hosts and host-selectivity of vector born organisms is crucial to understand basic molecular principles of host-pathogen relationship. Moreover, identification and characterization of the molecules circumventing host complement system is also important aspects to develop therapeutic or preventive approaches.

